# A mass spectrometry-based peptidomic dataset of the spermosphere in common bean (*Phaseolus vulgaris* L.) seeds

**DOI:** 10.1038/s41597-024-04044-y

**Published:** 2024-11-07

**Authors:** Chandrodhay Saccaram, Céline Brosse, Boris Collet, Delphine Sourdeval, Tracy François, Benoît Bernay, Massimiliano Corso, Loïc Rajjou

**Affiliations:** 1 Université Paris-Saclay, INRAE, AgroParisTech, Institut Jean-Pierre Bourgin for Plant Sciences (IJPB), 78000 Versailles, France; 2 Plateforme Proteogen, US EMerode, Université de Caen Normandie, 14000 Caen, France

**Keywords:** Plant signalling, Peptides

## Abstract

The spermosphere, a dynamic microenvironment surrounding germinating seeds, is shaped by the complex interactions between natural compounds exuded by seeds and seed-associated microbial communities. While peptides exuded by plants are known to influence microbiota diversity, little is known about those specifically exuded by seeds. In this study, we characterised the peptidome profile of the spermosphere for the first time using seeds from eight genotypes of common bean (*Phaseolus vulgaris*) grown in two contrasting production regions. An untargeted LC-MS/MS peptidomic analysis revealed 3,258 peptides derived from 414 precursor proteins of common bean in the spermosphere. This comprehensive peptidomic dataset provides valuable insights into the characteristics of peptides exuded by common bean seeds in the spermosphere. It can be used to identify peptides with potential antimicrobial or other biological activities, advancing our understanding of the functional roles of seed-exuded peptides in the spermosphere.

## Background & Summary

During germination, seeds release a complex mixture of organic and inorganic compounds into their surrounding environment, leading to the formation of a localised microenvironment known as the spermosphere, which is defined as the zone of increased microbial activity around germinating seeds^1,2^. While plant exudates have been extensively characterised and their roles in plant-microorganism interactions explored in other plant compartments, such as the rhizosphere^3,4^, seed exudates have received comparatively less attention. Similar to the interactions in the rhizosphere driven by root exudates, seed exudates during germination are likely instrumental in shaping the spermosphere microbiome^5^. A previous study characterising the proteome of germinating *Lupinus albus* seeds found that seed-exuded proteins are involved in plant defense mechanisms^6^. Indeed, proteins have long been recognised as playing crucial roles in plant immunity^7^.

Similar to proteins, several studies have characterised peptides that possess antimicrobial activities^8^. Although the seed peptidome has received limited attention, storage proteins (important reservoirs of potential peptides) are abundantly accumulated in seeds^9^. One study demonstrated that anti-microbial peptides (AMPs) are released by *Macadamia integrifolia* seeds during germination^10^, confirming the presence of seed-exuded peptides in the spermosphere. Beyond their role as antimicrobial compounds, plant-exuded peptides may also serve as a nutritional source for microorganisms^11^. This raises important questions about the composition, activities, and functions of seed-exuded peptides in the spermosphere. The current study aims to characterise peptides exuded by germinating seeds in the spermosphere, using common bean as a model system.

Common bean (*Phaseolus vulgaris* L.) is a widely consumed grain legume known for its high nutritional value and protein content in seeds^12,13^. As a legume species, common bean engages in mutually beneficial relationships with symbionts, mediated by complex molecular signalling between the host and rhizobial microsymbionts^14^. While nodulation in leguminous plants and the signalling processes involved in shaping the rhizosphere microbiome have been relatively well studied^14–17^, there is limited information on the early phase of the plant life cycle, particularly during seed germination. Previous research has shown that the common bean seed microbiome undergoes shifts in both abundance and community structure throughout germination^18^. This suggests the hypothesis that seeds may selectively influence their microbiome, at least partially, through exudates released during germination. Given that antimicrobial peptides have been found in seed exudates^10^ and considering that plant-exuded peptides can also serve as a nutrient source for microorganisms^11^, the protein-rich common bean seed provides an ideal model for exploring the diversity of peptides exuded in the spermosphere during germination.

In this study, we investigated the peptide composition of the spermosphere during the germination of common bean seeds under *in vitro* conditions. Our focus was on the peptides exuded by seeds over a 24-hour period. We analysed the peptidome profile in the spermosphere of eight different common bean genotypes, sourced from plants cultivated in two distinct geographical regions in France. The objectives were to (i) explore the peptidome landscape in the spermosphere and (ii) highlight the differences among the genotypes. Seeds were imbibed in sterile water at four time intervals (2, 8, 16, and 24 hours), and the surrounding water for each time interval was collected and pooled. Untargeted LC-MS/MS peptidomic analysis was then performed to assess the diversity of peptides in the pooled spermosphere.

## Methods

### Biological materials

**Fig. 1:**
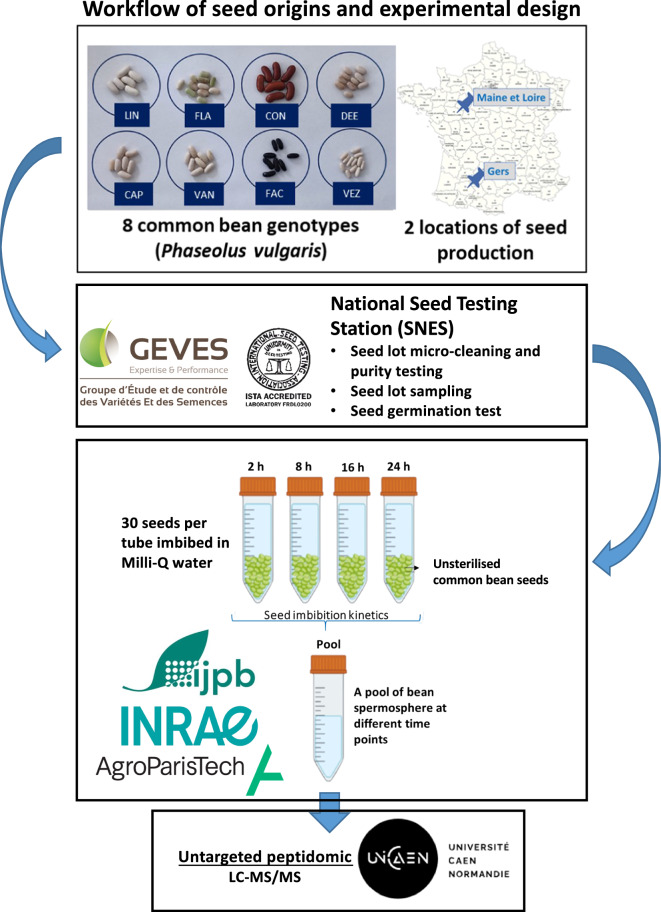
Workflow of experimental design for spermosphere collection and analysis. Spermosphere collection involved imbibing 30 unsterilised seeds per sample at four distinct time intervals: 2, 8, 16, and 24 hours. The surrounding water of the germinating seeds, representing the spermosphere, was collected at each time point and pooled for subsequent peptidomic analysis.

Seeds were harvested in 2020 from eight different common bean genotypes (French commercial cultivars of *Phaseolus vulgaris* L.), cultivated at two FNAMS field experimental stations: Gers in the southwest of France (43°57′25.2″N 0°23′31.7″E) and Maine-et-Loire (47°28′13.9″N 0°23′40.3″W) in the Loire Valley. The selected genotypes (i.e. VEZ, CON, CAP, FAC, FLA, LIN, DEE, and VAN) were chosen to represent the highest diversity among breeding programs and qualitative seed traits in France, as determined by the seed company Vilmorin-Mikado SAS (Limagrain group) (Fig. 1).

### Spermosphere production and collection

The experimental design for the collection of seed exudates in the spermosphere is illustrated in Fig. 1. This study characterised the composition of peptides exuded by seeds in the spermosphere and evaluated the impact of seed production location and genetic background on these exuded peptides. Each seed lot corresponded to seeds from a single genotype produced at one location. Three independent biological replicates were performed for each seed lot, resulting in a total of 48 samples (i.e. three replicates of eight genotypes produced at two locations).

To collect spermosphere samples from germinating seeds, 30 unsterilised seeds were used for each sample and imbibed at four distinct time intervals: 2, 8, 16, and 24 hours. The weight of the 30 seeds was determined before placing them in a 50 mL Falcon tube. The seeds were then imbibed with 1.5 times their total weight in sterile Milli-Q water. Imbibition was carried out with the samples (including blanks) placed on a platform shaker, agitated at 70 rpm, and maintained at 25 °C (Novotron INFORS AG, CH-4103 Bottmingen). The spermosphere, consisting of the water surrounding the germinating seeds, was collected at each time interval and pooled (250 µL per time interval) in 2 mL Eppendorf tubes for each sample. A blank sample of sterile Milli-Q water in a 50 mL Falcon tube was also included in the analyses.

The pooled spermosphere samples collected during the germination time course represent the interface between the seed and its environment. Each sample (1 mL) along with the blank was immediately flash-frozen in liquid nitrogen and stored at −80 °C for subsequent analysis.

### Extraction of peptides from spermosphere and untargeted LC-MS/MS peptidomic analysis

**Fig. 2:**
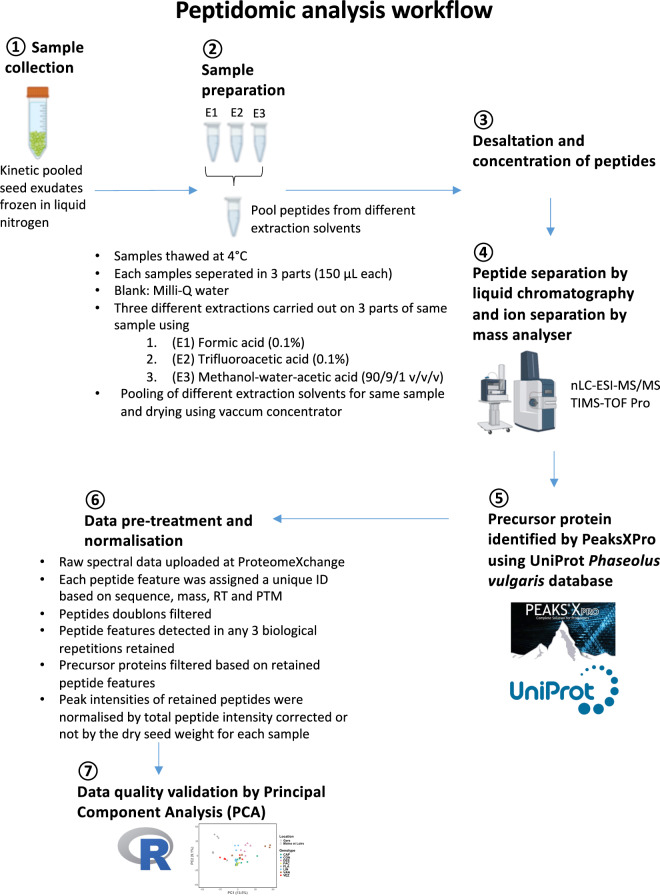
Peptidomic analysis workflow. This pipeline illustrates the steps involved in analysing the peptidome from the spermosphere of germinating common bean seeds.

The frozen pools of each spermosphere samples were thawed at 4 °C. Each sample (parent samples) were subsequently separated into three equal parts of 150 µL (daughter samples) in 2 mL Eppendorf tubes. To maximise peptide extraction diversity, three different extraction solvents were used for each daughter sample. Specifically, 1350 µL of the following solvents were added separately to each daughter sample: (i) 0.1% formic acid, (ii) 0.1% trifluoroacetic acid, and (iii) a methanol-water-acetic acid mixture (90/9/1 v/v/v). The daughter samples with their respective extraction solvents were stirred at 24 rpm on a rotator (Mini LabRoller™ Rotator, LabNet) at 4 °C for 30 minutes. Following extraction, the samples were centrifuged at 10,000 g to separate the soluble peptides, which were found in the supernatant, from the insoluble components (precipitate). The supernatants from the daughter samples were pooled to create a combined peptide extract for each parent sample, which was then dried using a SpeedVac vacuum concentrator (Thermo Scientific™ SPD 111 V) (Fig. 2).

Before nano-LC fragmentation analysis, peptide samples were desalted and concentrated using a µC18 Omix (Agilent). Briefly, the dried peptide samples were resuspended in 40 µL of 0.1% formic acid. These resuspended samples were then loaded onto a µC18 Omix column for desalting and concentration and subsequently eluted with 80% acetonitrile containing 0.1% formic acid. The eluted peptides were dried using a SpeedVac vacuum concentrator. The dried, concentrated peptide samples were then resuspended in 10 µL of 0.1% formic acid. One microliter of this resuspended peptide extract was injected for LC-MS/MS analysis. Chromatographic separation was performed using a NanoElute (Bruker Daltonics) ultra-high-pressure nano flow chromatography system. Peptide samples were first concentrated on a C18 PepMap 100 (5 mm × 300 µm i.d.) precolumn (Thermo Scientific) and then separated at 50 °C on a reversed-phase Reprosil column (25 cm × 75 µm i.d.) packed with 1.6 µm C18-coated porous silica beads (Ionopticks). The mobile phases were composed of 0.1% formic acid in 99.9% water (v/v) (Phase A) and 0.1% formic acid in 99.9% acetonitrile (v/v) (Phase B). The nanoflow rate was set at 300 nL/min, with the following gradient profile: from 2% to 15% Phase B within 15 minutes, increasing to 25% Phase B within 10 minutes, then to 37% Phase B within 12 minutes, followed by a decrease to 9% Phase B within 7 minutes and re-equilibration.

MS experiments were conducted on a TIMS-TOF Pro mass spectrometer (Bruker Daltonics) equipped with a modified nano electrospray ion source (CaptiveSpray, Bruker Daltonics). Ionisation was typically achieved with a spray voltage of 1600 V and a capillary temperature of 180 °C. MS spectra were acquired in positive mode over a mass range of 100 to 1700 m/z. The mass spectrometer operated in Parallel Accumulation–Serial Fragmentation (PASEF) mode without exclusion of singly charged peptides. A total of 10 PASEF MS/MS scans were performed over a 1.25-second cycle. The mass spectrometry proteomics raw spectral data have been deposited in the ProteomeXchange Consortium (https://proteomecentral.proteomexchange.org)^19^ via the iProX partner repository^20,21^, with the dataset identifier **PXD051625**^22^.

### Peptide identification

Peptide identification was performed using PEAKS XPro software (Bioinformatic Solutions Inc., CA) against an updated version of the UniProt *Phaseolus vulgaris* database (Proteome ID: UP000000226; The UniProt Consortium, 2022) (Fig. 2). The following modifications were included: methionine oxidation, N-terminal acetylation, deamidation, and carbamylation. The “no enzyme” parameter was selected for the analysis. Mass accuracy was set to 20 ppm for MS mode and 0.05 Da for MS/MS mode. Data were filtered with a false discovery rate (FDR) of 5%, and protein redundancy was eliminated based on proteins being evidenced by the same set or a subset of peptides.

### Data pre-treatment, normalisation and statistical analysis

**Fig. 3:**
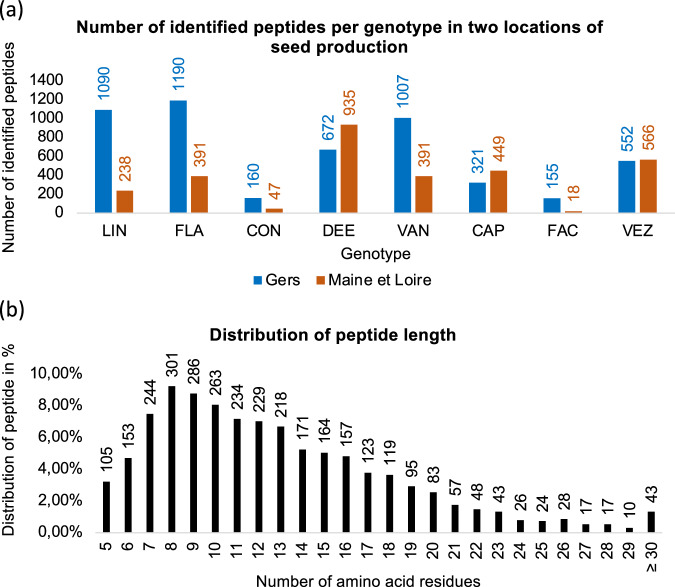
Characteristics of peptides in the spermosphere of germinating common beans. **(a)** Histograms displaying the number of peptides identified in the spermosphere for each common bean genotype produced in two different regions. Only peptides identified across all three biological replicates were included for each genotype and location. **(b)** Histogram showing the distribution of peptide lengths in the spermosphere of eight common bean genotypes from two production regions.

Each detected peptide ion feature was assigned a unique identifier based on its sequence, mass, retention time (RT), and post-translational modifications (PTMs). Peptide duplicates were then filtered out. Only peptide features detected in all three biological replicates were retained for further analysis. Precursor proteins were filtered based on the retained peptide features. Peak intensities of the retained peptides and proteins were normalised either by the sum of all peptide intensities of each sample or by the sum of all peptide intensities and corrected by the dry weight of 30 seeds. (Fig. 3). The complete lists of peptides, including both unfiltered and filtered datasets from the analysis, are publicly available in the *Recherche Data Gouv* multidisciplinary repository^23^ at https://entrepot.recherche.data.gouv.fr/dataverse/inrae, with the unique 10.57745/8CC18X^24^.

Principal Component Analysis (PCA) was performed on the peptidomic dataset using R (v.4.2.1). For peptide features that were detected but not quantified (peak intensities of 0), values were replaced by the smallest peak intensity in the dataset divided by 20. Peptide features that were not detected (empty cells) were assigned a peak intensity of 0. Two normalisation approaches were applied to the filtered peptide datasets. The first approach involved normalising each peptide peak intensity by the sum of all peptide intensities per sample. The second approach involved normalising each peptide intensity by the sum of all peptide intensities, with an additional correction for the aggregate dry weight of seeds per sample. Principal Component Analyses (PCA) were then generated from the normalised datasets (Fig. 7a,b).

## Data Records

The mass spectrometry proteomics raw spectral data is available at the ProteomeXchange online platform (https://proteomecentral.proteomexchange.org) via the iProX partner repository, under the dataset identifier **PXD051625**^22^. The complete lists of peptides from the analysis are available in the *Recherche Data Gouv* multidisciplinary repository at https://entrepot.recherche.data.gouv.fr/dataverse/inrae, with the unique 10.57745/8CC18X^24^.

The peptidomic datasets of the spermosphere samples from eight common bean genotypes produced at two locations, analysed by the Proteogen platform (University of Caen Normandy; https://www.unicaen.fr/laboratoire/proteogen/), are available under the name “Common_Bean_Spermosphere_Peptidomic_Datasets”^24^. The zip archive includes eight files, as detailed in Table 1.

**Table 1:** Data files from peptidomic data acquisition and pre-processing of spermosphere samples from germinating common beans.

File name	Explanation
Raw peptide dataset.xlsx	The file contains peptide features identified during the peptidomic analysis, including metrics such as protein precursor accessions (since peptides can originate from different protein precursors), peptide sequences, mass, m/z (mass-to-charge ratio), retention time (RT), peptide length, -10logP score, and raw peak intensities of each peptide sequence for each sample. For several peptide feature ions, peak intensities are recorded as 0, indicating that the peptide features were detected but not quantified. Peptide features that were not detected in samples are indicated by empty cells.
Raw precursor protein dataset.xlsx	The file contains precursor proteins of the identified peptides, including unique protein identifiers, protein accessions from the Uniprot database for *Phaseolus vulgaris*, protein descriptions, -10LogP identification scores, and peak intensities across samples. For several precursor proteins, peak intensities are recorded as 0, indicating that the precursor proteins were detected but not quantified. Precursor proteins that were not detected in samples are indicated by empty cells.
Filtered peptide dataset.xlsx	The file contains the final processed and filtered peptidome dataset. The peptidome dataset underwent preprocessing as follows: each peptide ion feature was assigned a unique identifier based on its amino acid sequence, retention time (RT), mass, and post-translational modifications (PTMs). Peptide duplicates were filtered out to remove redundant entries. Only peptide features detected in at least three biological replicates were retained for further analysis. A total of 3,258 peptide features were identified across the 48 samples in the common bean spermosphere.
Filtered precursor protein dataset.xlsx	The file contains the final processed and filtered precursor protein dataset. The preprocessing of the proteome dataset was conducted as follows: protein precursors were retained based on the corresponding retained peptides. When protein isoforms were encountered (i.e., proteins belonging to the same protein group), only one isoform was retained. A total of 414 protein precursors were identified.
Filtered peptide normalised dataset 1.csv	The file contains the normalised peak intensity data of the filtered peptidome dataset. Peptide features that were detected but not quantified (peak intensities of 0) were replaced with the smallest peak intensity value of the dataset divided by 20. Peptide features that were not detected (empty cells) were replaced with a peak intensity of 0. Each peptide peak intensity was then normalised by the sum of all peptide intensities for each sample. The normalised dataset was used for Principal Component Analysis (PCA) to assess the quality of the data (Fig. 7a).
Filtered peptide normalised dataset 2.csv	The file contains the normalised peak intensity data of the filtered peptidome dataset. Peptide features that were detected but not quantified (peak intensities of 0) were replaced with the smallest peak intensity value of the dataset divided by 20. Peptide features that were not detected (empty cells) were replaced with a peak intensity of 0. Each peptide intensity was then normalised by the sum of all peptide intensities per sample and further corrected by the aggregate dry weight of seeds for each sample. The normalised dataset was used for Principal Component Analysis (PCA) to assess the quality of the data (Fig. 7b).
Metadata.xlsx	The file contains the metadata of the experiment.
PCA Script.R	R code used for Principal Component Analysis (PCA).

This table lists the eight files associated with the peptidomic data acquisition and pre-processing for spermosphere samples from eight common bean genotypes produced in two locations. Details on the content of each file and the data processing steps applied (if any) are provided.

The “Raw peptide dataset.xlsx” file contains a list of all peptide features identified during the peptidomic analysis, while the “Raw precursor protein dataset.xlsx” file includes the precursor proteins (protein accessions from the UniProt database of *Phaseolus vulgaris*) corresponding to the identified peptides^24^. Details about the content of these files are provided in Table 1.

**Fig. 4:**
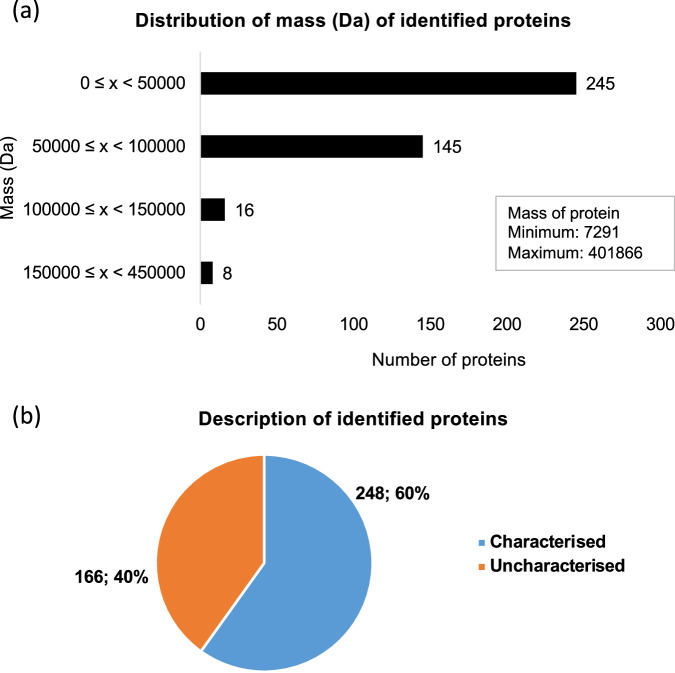
Characteristics of precursor proteins in the spermosphere of germinating common beans. **(a)** Histogram showing the mass distribution of precursor proteins associated with identified peptides in the spermosphere of eight common bean genotypes from two production regions. **(b)** Proportion of characterised precursor proteins identified through a search in the *Phaseolus vulgaris* UniProt database.

The “Filtered peptide dataset.xlsx” and “Filtered precursor protein dataset.xlsx” files contain the filtered and processed data from the untargeted peptidomic analysis, respectively^24^. The manual preprocessing steps used to generate these files are summarised in Table 1. A total of 3,258 peptide features were identified across the 48 common bean spermosphere samples, with substantial variability in the identified peptides observed across the eight genotypes produced in the two distinct locations (Fig. 3a). The identified peptide sequences ranged in length from 5 to 48 amino acids (Fig. 3b), and their masses ranged from 429.2587 Da to 4493.0254 Da. These peptides were derived from 414 precursor proteins, with precursor protein masses ranging from 7,291 Da to 401,866 Da (Fig. 4a). Most of the precursor proteins (60%) were annotated and had descriptions in the UniProt database for *Phaseolus vulgaris* (Proteome ID: UP000000226; The UniProt Consortium, 2022) (Fig. 4b).

The “Filtered peptide normalised dataset 1.csv” and “Filtered peptide normalised dataset 2.csv” files contains the normalised peak intensities of the data from the “Filtered peptide dataset.xlsx”^24^. The normalisation steps are described in detail in Table 1. Principal Component Analysis (PCA) was performed on this normalised peptidomic dataset (Fig. 7).

The “Metadata.xlsx” file provides detailed information on the 48 common bean spermosphere samples analysed in the experiment^24^.

## Technical Validation

**Fig. 5:**
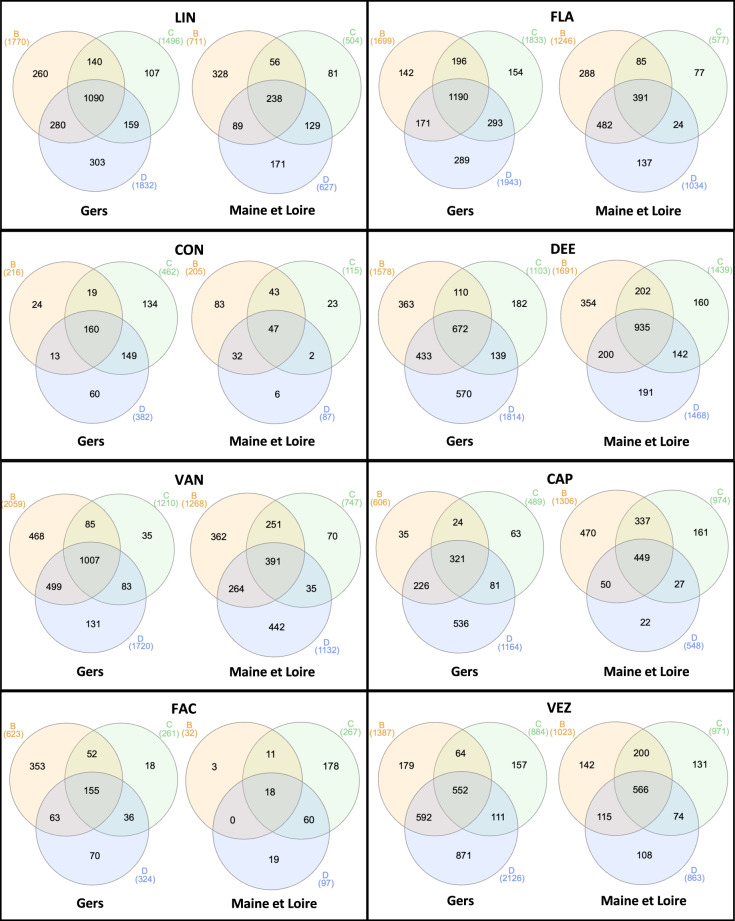
Pre-processing of peptidome dataset. Venn diagrams showing the overlap of identified peptides in biological replicates (B, C, and D) for each genotype at each production location. Only peptides identified in all three biological replicates at a given location were retained.
- Peptide and protein identification significance scores ensuring dataset robustness
  In mass spectrometry-based peptidomic and proteomic studies, the -10logP score (a.k.a. the significance score) is used to assess the confidence of peptide identifications^25^. PeaksXPro calculates this score based on a p-value (probability score P), which helps in interpreting peptide scoring for identification purposes^25^. The p-value reflects the probability that a false identification in the search yields the same or a better matching score. The -10logP score, which is the negative base-10 logarithm of this probability, quantifies the likelihood that a peptide-spectrum match (PSM) is a genuine match rather than a random occurrence. A higher -10logP value indicates a more significant match. For example, a p-value of 0.05 corresponds to a -10logP score of 13, while a p-value of 0.01 corresponds to a -10logP score of 20. In our study, peptides and protein features identified in the spermosphere of germinating common bean seeds have -10logP scores^25^ greater than 15, indicating a high level of confidence in their identification and thus ensuring the robustness of our datasets (Fig. 6a,b).

- Peptides identified in all three biological replicates
  To ensure the robustness of the peptidomic dataset, only peptide features that were consistently identified in all three biological replicates of each genotype were included. This approach was adopted to enhance the reliability and reproducibility of the dataset by focusing exclusively on peptides that appeared consistently across all replicates, thereby minimising bias from technical variations (Fig. 5).

**Fig. 6:**
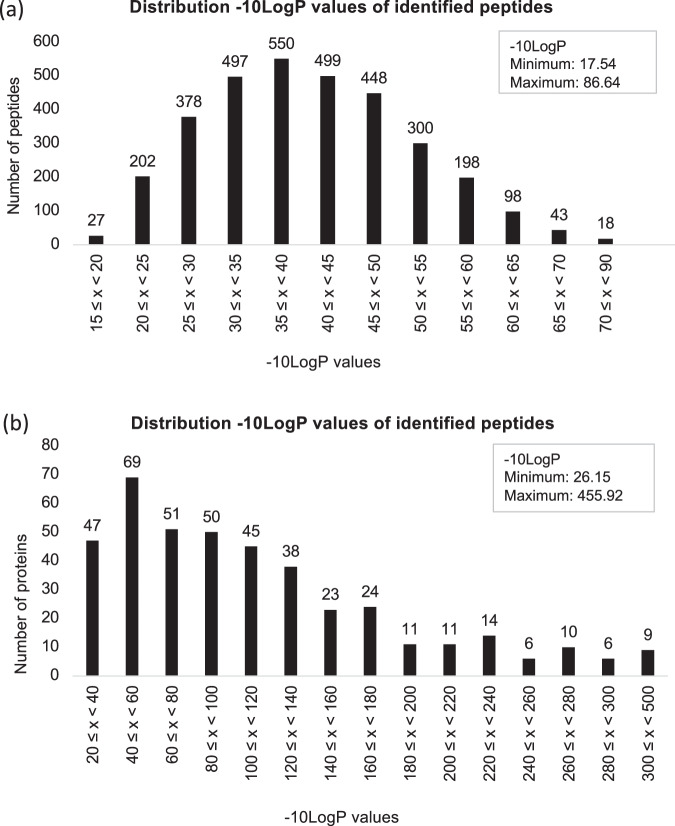
Peptides and protein identification scores. Histograms showing the distribution of the -10logP scores (a.k.a. significance scores), which measure the confidence in peptide and protein identifications. **(a)** Histogram of significance scores for retained identified peptides. **(b)** Histogram of significance scores for retained protein precursors.
- Principal Component Analysis (PCA)

**Fig. 7:**
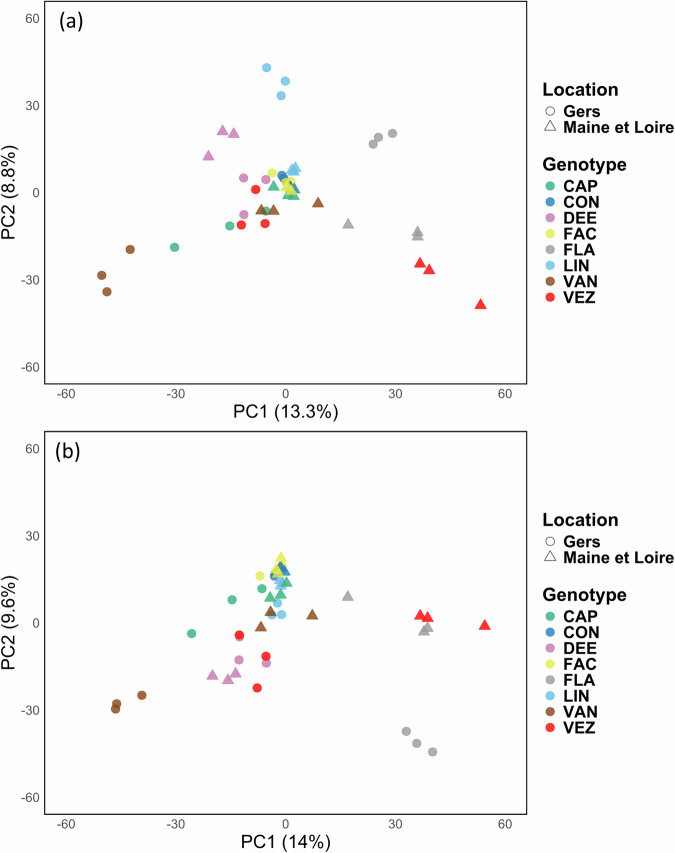
Principal component analysis (PCA) of peptide intensities. PCA is based on normalised peak intensities of identified peptides. Different colours represent the eight common bean genotypes, while different shapes indicate the seed production locations (**a**). Single peptide intensity normalised by the sum of all peptide intensities per sample (**b**). Single peptide intensity normalised by both the sum of all peptide intensities and aggregate dry weight of seeds per samples.

PCA based on normalised peptide intensities effectively grouped biological replicates together, demonstrating a consistent clustering across all samples independently of the normalisation approach (Fig. 7a,b). Compared to other genotypes, the analysis revealed that the VAN, FLA and VEZ genotypes exhibited a more distinct peptidome composition between samples produced from the two locations (“Gers” and “Maine et Loire”).

## Usage Notes

During seed germination, molecules and macromolecules are released into the surrounding environment^2^. Given that proteins are highly accumulated in seeds^26^, it is anticipated that they are among the compounds exuded. However, there is limited research on proteins in this context^27^, and consequently, there is scant information regarding the peptides and their origins in the spermosphere.

Here, we have characterised for the first time the diversity of peptides in the spermosphere using eight different common bean genotypes produced in two distinct locations. Peptides are known to play diverse roles in plant growth, development, and stress responses^28^. Additionally, their specific structures are thought to confer a wide range of bioactivities^28^. Our dataset, which details the peptide diversity in the spermosphere, may contribute to the scientific community’s understanding of how seeds interact with their environment during germination through peptide-mediated processes^29,30^.

## Data Availability

The Principal Component Analysis R script, named “PCA Script.R”, is available at *Recherche Data Gouv* public repository with unique 10.57745/8CC18X^24^. Analyses were performed using R v4.2.1.

## References

[1] Verona, O. [The spermosphere]. *Ann. Inst. Pasteur* **95**, 795–798 (1958). 13617727

[2] Nelson, E. B. The seed microbiome: Origins, interactions, and impacts. *Plant Soil* **422**, 7–34 (2018).

[3] Sasse, J., Martinoia, E. & Northen, T. Feed Your Friends: Do Plant Exudates Shape the Root Microbiome? *Trends Plant Sci.* **23**, 25–41 (2018). 29050989 10.1016/j.tplants.2017.09.003

[4] Bai, B. *et al*. The root microbiome: Community assembly and its contributions to plant fitness. *J. Integr. Plant Biol.* **64**, 230–243 (2022). 35029016 10.1111/jipb.13226

[5] Yu, P. *et al*. Plant flavones enrich rhizosphere Oxalobacteraceae to improve maize performance under nitrogen deprivation. *Nat. Plants* **7**, 481–499 (2021). 33833418 10.1038/s41477-021-00897-y

[6] Scarafoni, A. *et al*. The proteome of exudates from germinating Lupinus albus seeds is secreted through a selective dual-step process and contains proteins involved in plant defence. *FEBS J.* **280**, 1443–1459 (2013). 23332028 10.1111/febs.12140

[7] Jain, M., Amera, G. M., Muthukumaran, J. & Singh, A. K. Insights into biological role of plant defense proteins: A review. *Biocatal. Agric. Biotechnol.* **40**, 102293 (2022).

[8] Li, J., Hu, S., Jian, W., Xie, C. & Yang, X. Plant antimicrobial peptides: structures, functions, and applications. *Bot. Stud.* **62**, 5 (2021). 33914180 10.1186/s40529-021-00312-x PMC8085091

[9] Ayala-Niño, A. *et al*. Sequence Identification of Bioactive Peptides from Amaranth Seed Proteins (Amaranthus hypochondriacus spp.). *Molecules* **24**, 3033 (2019). 31438557 10.3390/molecules24173033 PMC6749583

[10] Marcus, J. P., Green, J. L., Goulter, K. C. & Manners, J. M. A family of antimicrobial peptides is produced by processing of a 7S globulin protein in Macadamia integrifolia kernels. *Plant J.* **19**, 699–710 (1999). 10571855 10.1046/j.1365-313x.1999.00569.x

[11] Hill, P. W., Farrell, M. & Jones, D. L. Bigger may be better in soil N cycling: Does rapid acquisition of small l-peptides by soil microbes dominate fluxes of protein-derived N in soil? *Soil Biol. Biochem.* **48**, 106–112 (2012).

[12] Los, F. G. B., Zielinski, A. A. F., Wojeicchowski, J. P., Nogueira, A. & Demiate, I. M. Beans (*Phaseolus vulgaris* L.): whole seeds with complex chemical composition. *Curr. Opin. Food Sci.* **19**, 63–71 (2018).

[13] Singh, N., Jain, P., Ujinwal, M. & Langyan, S. Escalate protein plates from legumes for sustainable human nutrition. *Front. Nutr.* **9**, 977986 (2022). 36407518 10.3389/fnut.2022.977986 PMC9672682

[14] Oldroyd, G. E. D., Murray, J. D., Poole, P. S. & Downie, J. A. The Rules of Engagement in the Legume-Rhizobial Symbiosis. *Annu. Rev. Genet.* **45**, 119–144 (2011). 21838550 10.1146/annurev-genet-110410-132549

[15] Servín-Garcidueñas, L. E. *et al*. Symbiont shift towards *Rhizobium* nodulation in a group of phylogenetically related *Phaseolus* species. *Mol. Phylogenet. Evol.* **79**, 1–11 (2014). 24952318 10.1016/j.ympev.2014.06.006

[16] Bag, S., Mondal, A., Majumder, A., Mondal, S. K. & Banik, A. Flavonoid mediated selective cross-talk between plants and beneficial soil microbiome. *Phytochem. Rev.* **21**, 1739–1760 (2022). 35221830 10.1007/s11101-022-09806-3 PMC8860142

[17] Lepetit, M. & Brouquisse, R. Control of the rhizobium–legume symbiosis by the plant nitrogen demand is tightly integrated at the whole plant level and requires inter-organ systemic signaling. *Front. Plant Sci.* **14**, 1114840 (2023). 36968361 10.3389/fpls.2023.1114840 PMC10033964

[18] Torres-Cortés, G. *et al*. Functional Microbial Features Driving Community Assembly During Seed Germination and Emergence. *Front. Plant Sci.* **9**, 902 (2018). 30008730 10.3389/fpls.2018.00902 PMC6034153

[19] Deutsch, E. W. *et al*. The ProteomeXchange consortium at 10 years: 2023 update. *Nucleic Acids Res.* **51**, D1539–D1548 (2022). 10.1093/nar/gkac1040 PMC9825490 36370099

[20] Ma, J. *et al*. iProX: an integrated proteome resource. *Nucleic Acids Res.* **47**, D1211–D1217 (2019). 30252093 10.1093/nar/gky869 PMC6323926

[21] Chen, T. *et al*. iProX in 2021: connecting proteomics data sharing with big data. *Nucleic Acids Res.* **50**, D1522–D1527 (2021). 10.1093/nar/gkab1081 PMC8728291 34871441

[22] Bernay, B. & Rajjou, L. Characterization of the peptidome in the spermosphere of common bean (*Phaseolus vulgaris*). *ProteomeXchange* https://identifiers.org/px:PXD051625 (2024).

[23] Dzalé Yeumo, E. Data INRAE – The Networked Repository. in *Research Data Sharing and Valorization* 63–75 10.1002/9781394163410.ch4 (John Wiley & Sons, Ltd, 2022).

[24] Saccaram, C., Bernay, B. & Rajjou, L. Untargeted peptidomic (LC-MS/MS) analysis of the spermosphere of eight common bean genotypes (*Phaseolus vulgaris* L.). DataGOUV 10.57745/8CC18X (2024).

[25] Zhang, J. *et al*. PEAKS DB: De Novo Sequencing Assisted Database Search for Sensitive and Accurate Peptide Identification. *Mol. Cell. Proteomics MCP* **11**, M111.010587 (2012). 22186715 10.1074/mcp.M111.010587 PMC3322562

[26] Fujiwara, T., Nambara, E., Yamagishi, K., Goto, D. B. & Naito, S. Storage proteins. *Arab. Book* **1**, e0020 (2002). 10.1199/tab.0020 PMC3243327 22303197

[27] Rocha, R. O. *et al*. Proteome of Soybean Seed Exudates Contains Plant Defense-Related Proteins Active against the Root-Knot Nematode *Meloidogyne incognita*. *J. Agric. Food Chem.* **63**, 5335–5343 (2015). 26034922 10.1021/acs.jafc.5b01109

[28] Tavormina, P., De Coninck, B., Nikonorova, N., De Smet, I. & Cammue, B. P. A. The Plant Peptidome: An Expanding Repertoire of Structural Features and Biological Functions. *Plant Cell* **27**, 2095–2118 (2015). 26276833 10.1105/tpc.15.00440 PMC4568509

[29] Marmiroli, N. & Maestri, E. Plant peptides in defense and signaling. *Peptides* **56**, 30–44 (2014). 24681437 10.1016/j.peptides.2014.03.013

[30] Ren, G., Zhang, Y., Chen, Z., Xue, X. & Fan, H. Research Progress of Small Plant Peptides on the Regulation of Plant Growth, Development, and Abiotic Stress. *Int. J. Mol. Sci.* **25**, 4114 (2024). 38612923 10.3390/ijms25074114 PMC11012589

